# Outcomes of Adults With Relapsed/Refractory Acute Myeloid Leukemia Treated With Venetoclax Plus Hypomethylating Agents at a Comprehensive Cancer Center

**DOI:** 10.3389/fonc.2021.649209

**Published:** 2021-03-11

**Authors:** Matthew E. Tenold, Benjamin N. Moskoff, David J. Benjamin, Rasmus T. Hoeg, Aaron S. Rosenberg, Mehrdad Abedi, Joseph M. Tuscano, Brian A. Jonas

**Affiliations:** ^1^ Department of Internal Medicine, Division of Hematology and Oncology, University of California Davis School of Medicine, Sacramento, CA, United States; ^2^ Pharmacy Department, University of California Davis School of Medicine, Sacramento, CA, United States; ^3^ Division of Hematology and Oncology, Department of Internal Medicine, University of California, Irvine Medical Center, Orange, CA, United States; ^4^ Veterans Administration Northern California Healthcare System, Sacramento, CA, United States

**Keywords:** acute myeloid leukemia, venetoclax (BCL-2 inhibitor), hypomethylating agent, relapsed/refractory, real-world data

## Abstract

Relapsed/refractory acute myeloid leukemia (AML) is a devastating disease with a poor prognosis and represents a major unmet medical need. We report on a real-world academic center experience of treating 25 patients with relapsed/refractory AML using venetoclax in combination with decitabine or azacitidine, which is not otherwise widely evaluated in the current literature. Our patients come from a large, socioeconomically and geographically diverse area including the majority of Northern California. Most had ELN Adverse Risk (52%) or Intermediate Risk (44%) AML, and most had an ECOG Performance Status of 1 (64%). Over half (52%) had prior hypomethylating agent exposure, and 40% had Secondary AML. We observed an overall response rate of 52%, with eight patients (32%) achieving composite complete remission. Median overall survival was 5.5 months, and for patients achieving composite complete remission this was 21.6 months. One-year estimated overall survival was 38%. Three patients were able to proceed directly to stem cell transplant for consolidation, and all three were alive at last follow-up, ranging 13.8–24.0 months. We found venetoclax in combination with hypomethylating agents to be well tolerated and potentially efficacious in securing long-term remissions for patients with relapsed/refractory AML.

## Introduction

Acute myeloid leukemia (AML) is the most common acute leukemia among adults. In 2020, 19,940 new cases and 11,180 deaths are estimated in the United States (1). Despite advances in therapy, prognosis remains poor, with 28% alive at 5 years after diagnosis (2). Treatment of older adults is challenging due to the high frequency of unfavorable disease characteristics, such as complex cytogenetics or adverse mutations, and less favorable patient characteristics, such as declining performance status and more frequent medical comorbidities (3, 4). In particular, relapsed/refractory (R/R) AML is associated with dismal outcomes, and up to 57% of patients experience primary refractory AML, relapse after CR, or death in the first 12 months after diagnosis (5).

B-cell lymphoma-2 (BCL-2) is a member of the BCL-2 family of anti-apoptotic mitochondrial proteins and has been shown to enhance AML cell survival and therapeutic resistance. Venetoclax is an oral selective BCL-2 inhibitor that has anti-AML and anti-leukemia stem cell activity as a monotherapy and acts synergistically when combined with hypomethylating agents (HMA) (6–9). Venetoclax was granted accelerated approval by the U.S. FDA in November 2018 for the frontline treatment of *de novo* AML in combination with an HMA (HMA/Ven) in patients ≥75 years or ineligible for standard chemotherapy. This decision was supported by early phase studies showing a composite complete remission [cCR: a composite of complete remission (CR) and complete remission with incomplete hematologic recovery (CRi)] of 61–73% (8, 10). The efficacy of HMA/Ven combinations in R/R AML is less clear. One retrospective report of treating R/R AML found a relatively low objective response rate (ORR: defined as the sum of CR, CRi, and MLFS) of 21% with median overall survival (OS) of 3 months (11). A second study of 21 patients reported an ORR of 28.6% and 3-month OS of 72% (12). A third study found a more promising ORR of 64%, with CR in 21% and CRi in 12% of patients, and 12-month OS of 53%; however, no responses were seen in patients with Secondary AML (sAML), and median follow-up was 6.5 months (13). A fourth study of 14 patients found an ORR of 35.7%, median OS of 4.7 months, and 12-month OS of 23.6% (14). Following these, a systematic review and meta-analysis of HMA/Ven in this role were performed, which included three of the aforementioned studies and four additional studies in a quantitative synthesis (15). They confirmed statistically significant heterogeneity among the data and reported an ORR of 38.7% and CR rate of 19.0% from this combined data for patients treated with VEN + HMA or low-dose cytarabine (LDAC). They also determined a median OS ranging from 3.0 to 6.6 months for this group of patients. Here we report the experience of the UC Davis Comprehensive Cancer Center (UCDCCC) in treating R/R AML with HMA/Ven.

## Methods

Following institutional review board approval, we retrospectively identified consecutive adult (≥18 years) patients with R/R AML who received HMA/Ven from January 1, 2014 to June 22, 2018 using an internal pharmacy database. Patients enrolled on a clinical trial were excluded. All patients were assessed for 2017 European Leukemia Net (ELN) genetic risk stratification (16). Treatment was based on a prior publication (17) using a 28-day venetoclax cycle with an HMA backbone consisting of decitabine 20 mg/m^2^ intravenously daily on days 1–5 or azacitidine 75 mg/m^2^ intravenously or subcutaneously daily on days 1–7. Venetoclax was given orally on days 1–28 for all patients in cycle 1. There were no patients concurrently receiving other anti-leukemic therapies, such as FLT3, IDH1, or IDH2 inhibitors. During this time period it was our standard to admit all patients, start antifungal prophylaxis, and begin a venetoclax ramp-up with the target dose adjusted for CYP3A4 interactions under inpatient monitoring. Efficacy outcomes included CR, CRi, cCR, morphologic leukemia free state (MLFS), ORR, and OS were based on the 2017 ELN Criteria (16). Events were evaluated using the Kaplan–Meier method and statistically significant differences (p ≤ 0.05) were determined using Fisher’s exact test and log-rank tests with Microsoft Excel^®^ (Microsoft^®^, Seattle, WA) and GraphPad^®^ (GraphPad Software^®^, La Jolla, CA).

## Results

**Figure 1:**
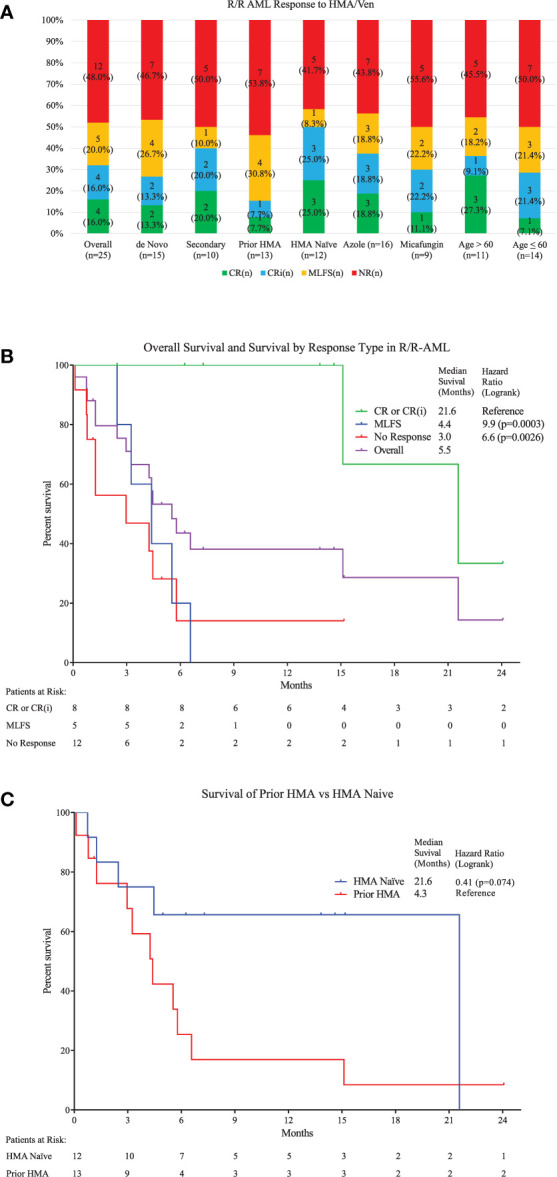
**(A)** Top: Response types to therapy are shown for the overall population and several subgroups with specific risk factors. No significant differences were seen between different risk groups (**Table 1**). Overall, 4 had CR (16%), 4 Cri (16%), and 5 MLFS (20%), for an ORR 52%. **(B)** Middle: Kaplan-Meier survival estimates are shown. A median follow-up of 14.6 months was seen (calculated by reverse-Kaplan-Meier). Median Overall Survival (OS) for the whole population was 5.5 (95% Cl, 2.9-21.6) months. Median OS for patients achieving cCR was 21.6 (95% Cl, 15.2-Not Reached) months, which was significantly longer than for those achieving MLFS at 4.4 (95% Cl, 2.4-6.6) months or no response at 3.0 (95% Cl, 0.8-5.8) months (Log-rank Chi^2^ 11.62, p<0.009). One-year estimated OS was 38% for the entire population (95% Cl, 18.4% - 57.5%). **(C)** Bottom: Median OS for patients who were HMA-naïve is compared to those with prior HMA use. The HMA-naïve group had a median OS of 21.6 (95% Cl, 1.3-Not Reached) months compared to 4.3 (95% Cl, 1.3-6.6) months for those with prior exposure either for MDS treatment (n=2) or prior AML treatment (n=10) (HR=0.40, 95% Cl=0.15 to 1.07.)

Twenty-five patients were identified (**Table 1**). The number of patients and response rates were similar between patients age ≤60 or age >60, *de novo* AML or sAML, prior HMA exposure or HMA naivety, ELN Genetic Risk, and azole or echinocandin prophylaxis. Eleven patients received prior HMA for AML. Four patients achieved CR (16%), four CRi (16%), and five MLFS (20%), for an ORR 52% and cCR 32% (**Figure 1A** and **Supplemental Table 1**). A median of two cycles [1–10] of HMA/Ven were given, with a median time to response of 1.6 months [0.8–5.1], or one cycle [1–3]. One patient with sAML from chronic myelomonocytic leukemia (CMML) achieved a CR, and one patient with sAML from primary myelofibrosis achieved a MLFS. Five (20%) patients had undergone hemopoietic stem cell transplant (HSCT) prior to treatment, with one achieving CRi and one MLFS. Next-generation sequencing data was available for 16 (64%) patients, and cCR was seen in 50% (two of four) of patients with IDH1 or IDH2, 60% (three of five) with FLT3-ITD, and 66% (two of three) with NPM1 mutations (**Supplemental Figure 1**).

**Table 1:** Patient characteristics.

Category	#, (%), [range]
Total Patients	25
Age	
Median Age	57 [25–86]
Age > 60	11 (44%)
Age ≤ 60	14 (56%)
Gender	
Male	14 (56%)
Female	11 (44%)
Race (Self-identified)	
Caucasian	16 (64%)
Asian	2 (8%)
Hispanic (non-Caucasian)	3 (12%)
African American	4 (16%)
ECOG Performance Status	
0	5 (20%)
1	16 (64%)
2	3 (12%)
3	1 (4%)
4	N/A
Pre-treatment Hematologic Parameters	
Median Serum Abs. Blasts (k/mm^3^)	0.10 [0–32,570]
Median Serum Abs. WBC (k/mm^3^)	1.75 [0–131.5]
Median Bone Marrow Blasts (% by Aspirate)	33.0% [6–95%]
AML Status	
De novo AML	15 (60%)
Secondary AML	10 (40%)
Prior MDS	6 (24%)
Prior CML	1 (4%)
Prior CMML	2 (8%)
Prior PMF	1 (4%)
2017 ELN risk stratification by genetics^14^	
Favorable	1 (4%)
Intermediate	11 (44%)
Adverse	13 (52%)
AML Status	
Refractory	12 (48%)
Relapsed	11 (44%)
Both	2 (8%)
Prior Treatment Exposures	
Median number of prior therapies	2 [1–6]
HMA Naïve	12 (48%)
Prior HMA*	13 (52%)
Decitabine	1 (4%)
Azacitadine	9 (36%)
Median number of prior HMA cycles before refractory	5 [1–13]
Prior Frontline “7+3”	18 (72%)
Other Prior Intense Chemotherapy (*e.g.* Clinical Trial, FLAG, MEC, CVD)	4 (16%)
HMA + Venetoclax Course Characteristics	
Median Cycles of Venetoclax	2 [1–10]
Median Months to Response	1.6 [0.8–5.1]
Median Cycles to Response	1 [1–3]
HSCT After Venetoclax	3 (12%)
Prior HSCT Before Venetoclax	5 (20%)
Antifungal Prophylaxis	
Azole	16 (64%)
Micafungin	9 (36%)

*Eleven patients had prior HMA for a diagnosis of AML.

After a median follow-up of 14.6 months, median OS was 5.5 (95% CI, 2.9–21.6) months (**Figure 1B** and **Supplemental Table 1**). Median OS for patients achieving cCR was 21.6 (95% CI, 15.2-not reached) months, which was significantly longer than for those achieving MLFS at 4.4 (95% CI, 2.4–6.6) months or no response at 3.0 (95% CI, 0.8–5.8) months (p < 0.0026). One-year estimated OS was 38% for the entire population. For patients obtaining cCR, MLFS or no response, estimated survival at one-year survival was 100, 0, and 14%, respectively. No difference was seen between the median survival of patients with *de novo* AML or sAML, azole or echinocandin prophylaxis, and age ≤60 or age >60. Being naïve to prior HMA use trended toward improved survival at 21.6 (95% CI, 1.3-not reached) months compared to 4.3 (95% CI, 1.3–6.6) months (HR = 0.411, p = 0.074) (**Figure 1C**). ELN Genetic Risk status did not significantly impact survival duration in this population (**Supplemental Figure 2**). Median duration of response was 14.7 (95% CI, 1.8-not reached) months. Median relapse free survival was 17.0 (95% CI, 3.0-not reached) months. Five of the 13 responders (38%) experienced relapse. Median time to relapse was 3.6 [3.0–17.0] months from start of therapy for these five patients, and they all expired with a median survival after relapse of 3.5 [0.8–11.0] months. Three of four patients who obtained a CR were able to proceed directly to HSCT for consolidation, and all three were alive at last follow-up [13.8–24.0 months]. One of these patients relapsed after HSCT, but obtained another CR with additional HMA/Ven and donor-lymphocyte infusion. Another patient who was refractory to HMA/Ven responded to another regimen and underwent HSCT. Of the nine (36%) patients alive at the end of the study period, four (44%) had undergone HSCT while five (56%) continued HMA/Ven maintenance.

Febrile neutropenia occurred in 10 (40%) patients. Prolonged pancytopenia (requiring a treatment delay of >14 days) was experienced by nine (36%). Nine (36%) patients were treated with micafungin with one (11.1%) experiencing a breakthrough fungal infection (BFI), and 16 (64%) received a broad-spectrum azole for prophylaxis with three (18.8%) BFIs. No patients had tumor lysis syndrome (TLS). Early mortality (death within 30 days of therapy initiation) occurred in 3 (12%) patients—one experienced a ventricular fibrillation cardiac arrest and was unable to be resuscitated, one developed an aspergillus pneumonia, and one had refractory AML. Two additional patients died within 60 days from pneumonia, for a total of five (20%) deaths within 60 days of treatment. None had achieved a response. Overall, 16 (64%) deaths were recorded. The most common cause was R/R AML in 10 (62.5%) patients followed by infection in four (25%).

## Discussion

The experience with HMA/Ven at UCDCCC expands on the published literature, demonstrating a well-tolerated, active regimen for patients with R/R *de novo* and sAML. Our ORR of 52% was favorable in the context of the previously reported range (21–64%) (11–15), which could relate to differences in the population included in our study. Unlike the prior literature (13), which showed a 0% response rate and 0% 6-month survival in patients with sAML, we found an ORR of 50%, which was not significantly different from our response rate in *de novo* AML. Additionally, 45% of our sAML patients were alive 12 months, and 22% survived to at least 24 months in this high-risk population. Recently, a non-randomized Phase II clinical trial included a subset of 55 (33%) R/R AML patients treated with decitabine and venetoclax and found a similar ORR (62%), median OS (7.8 months), and median duration of response (16.8 months) in this R/R subgroup (18). Our retrospective findings are generally more aligned with these prospective data than with the prior retrospective literature.

Prior HMA exposure trended towards worse response and survival when compared to HMA-naivety, where we saw an ORR among those with prior HMA exposure of six (46.2%), including one (7.7%) CR and one (7.7%) CRi for a cCR of two (15.4%). These responders had a median OS of 6.1 (95% CI, 3.2-not reached) months for those having ORR. Our patient with CRi has survived 24 months and was alive at last follow-up and our patient with CR survived 15 months, but unfortunately expired 1.6 months after undergoing Allo-SCT after experiencing cardiac complications while recovering from transplant. This population is not widely reported due to exclusion of patients with prior HMA exposure in clinical trials; however, it was also evaluated in another retrospective review which saw a cCR of 43%, and median OS of 10.8 months in those who achieved remission (19). Based on this report and our own experience, the depth of response appears particularly important in this population, which we also saw for our population overall. From the vantage of a relatively long median follow-up period, the depth of response significantly impacted survival for our patients. The largest benefit was seen after CR or CRi, with 100% survival at 12 months for this group, and an estimated median OS of 21.6 months. Survival was short after an MLFS response, and at 6 months more of the non-responding patients were found to be alive than patients with MLFS from HMA/Ven. Detailed review of these 5 patients with MLFS response revealed that inability to bridge to transplant contributed to these poor outcomes. Relapse of AML was the primary cause of death in three patients. Two of these patients were not candidates for transplant due to age and medical co-morbidities, while a 3^rd^ relapsed while undergoing transplant work-up and did not consider to further re-induction. A 4^th^ patient with MLFS had substantial social barriers to safe transplant, and developed aspergillus pneumonia 6 months after MLFS response. The 5^th^ patient experienced a massive, fatal GI bleed while being considered for transplant. The use of HMA/Ven as a bridge to HSCT was successful in several patients with deeper responses who remained in remission at the end of follow-up. This regimen may represent a viable means of low-intensity re-induction prior to HSCT consolidation. No TLS was observed, possibly due to intensive prophylaxis and monitoring. Relatively few BFI occurred, suggesting that prophylaxis with an echinocandin or broad-spectrum azole may be beneficial without impacting survival. Limitations of this study include its retrospective design, the limited number of patients, and the rapidly evolving nature of AML diagnostics and treatment during the time-period evaluated.

In conclusion, in this retrospective, real-world study, we found HMA/Ven to be an active and well-tolerated regimen for R/R AML patients. Based on our results here, we believe this combination represents a viable salvage regimen in R/R AML for patients whom a clinical trial is not available, for whom other intensive chemotherapy regimens have already failed, or whom would not likely otherwise tolerate intensive chemotherapy due to their age or performance status. The role of HMA/Ven in the treatment of R/R AML patients should be further confirmed and optimized in additional prospective controlled clinical trials and by combining real-world data from multiple healthcare systems.

## Data Availability Statement

The original contributions presented in the study are included in the article/**Supplementary Material**. Further inquiries can be directed to the corresponding author.

## Ethics Statement

The studies involving human participants were reviewed and approved by University of California, Davis Institutional Review Board. Written informed consent for participation was not required for this study in accordance with the national legislation and the institutional requirements.

## Author Contributions

MT, BM, and BJ conceptualized and designed the study. MT, BM, and DB acquired the data. MT, BM, DB, RH, AR, MA, JT, and BJ analyzed and interpreted the data. MT, BM, DB, RH, AR, MA, JT, and BJ wrote, reviewed, and/or revised the manuscript. All authors contributed to the article and approved the submitted version.

## Funding

This project and publications fees are supported by institutional funds.

## Conflict of Interest

AR has received research funding from Amgen. He has participated in Speakers Bureaus for Janssen and Millenium-Takeda. He has served in a consulting/advisory role for Seattle Genetics and Karyopharm. MA has participated in Speakers Bureaus for AbbVie, Celgene, BMS, and Gilead. JT has received research funding from Celgene, Novartis, Achrotech, Pharmacyclics, Genentech, and Takeda. He has received honoraria from Celgene, Amgen and Seattle Genetics. Dr. Jonas has served in a consulting/advisory role for AbbVie, Amgen, Celgene, Genentech/Roche, GlycoMimetics, Jazz, Takeda, Tolero, and Treadwell. He has received travel support from AbbVie, Amgen, and GlycoMimetics. He has received research funding to his institution from 47, AbbVie, Accelerated Medical Diagnostics, Amgen, AROG, Celgene, Daiichi Sankyo, Esanex, F. Hoffmann-La Roche, Forma, Genentech/Roche, GlycoMimetics, Hanmi, Incyte, Jazz, LP Therapeutics, Pfizer, Pharmacyclics, and Sigma Tau.

The remaining authors declare that the research was conducted in the absence of any commercial or financial relationships that could be construed as a potential conflict of interest.
