# Supplementary material for: Outcomes of Adults With Relapsed/Refractory Acute Myeloid Leukemia Treated With Venetoclax Plus Hypomethylating Agents at a Comprehensive Cancer Center
Source: Front Oncol. 2021 Mar 11;11:649209. doi: 10.3389/fonc.2021.649209 (PMC7991747; doi:10.3389/fonc.2021.649209)
Supplement: Supplementary file 3 [file Table_1.docx]

| **Supplemental Table 1: Responses & Survival Outcomes** | | | | | | | | | | | | | | | | |
| --- | --- | --- | --- | --- | --- | --- | --- | --- | --- | --- | --- | --- | --- | --- | --- | --- |
|  | **Total** | **ORR(n)** | **ORR (%)** | **P** | **CR(n)** | **CR (%)** | **CRi(n)** | **CRi(%)** | **MLFS(n)** | **MLFS(%)** | **NR(n)** | **NR(%)** | **Median OS (Months)** | **Hazard Ratio** | **95% CI** |  |
| **Overall** | **25** | **13** | **52.0%** |  | **4** | **16.0%** | **4** | **16.0%** | **5** | **20.0%** | **12** | **48.0%** | **5.5** |  |  |  |
| de Novo | 15 | 8 | 53.3% | 1.0000 | 2 | 13.3% | 2 | 13.3% | 4 | 26.7% | 7 | 46.7% | 5.8 | 0.84 | 0.31 to 2.28 |  |
| Secondary | 10 | 5 | 50.0% | NS | 2 | 20.0% | 2 | 20.0% | 1 | 10.0% | 5 | 50.0% | 4.5 | 1.18 | 0.43 to 3.19 |  |
| Prior HMA | 13 | 6 | 46.2% | 0.6951 | 1 | 7.7% | 1 | 7.7% | 4 | 30.8% | 7 | 53.8% | 4.4 | 2.49 | 0.93 to 6.64 |  |
| HMA Naïve | 12 | 7 | 58.3% | NS | 3 | 25.0% | 3 | 25.0% | 1 | 8.3% | 5 | 41.7% | 21.6 | 0.40 | 0.15 to 1.07 |  |
| Azole | 16 | 9 | 56.3% | 1.0000 | 3 | 18.8% | 3 | 18.8% | 3 | 18.8% | 7 | 43.8% | 4.3 | 1.25 | 0.43 to 3.56 |  |
| Micafungin | 9 | 5 | 55.6% | NS | 1 | 11.1% | 2 | 22.2% | 2 | 22.2% | 5 | 55.6% | 5.8 | 0.80 | 0.28 to 2.27 |  |
| Age > 60 | 11 | 6 | 54.5% | 1.0000 | 3 | 27.3% | 1 | 9.1% | 2 | 18.2% | 5 | 45.5% | 5.5 | 1.7 | 0.62 to 4.75 |  |
| Age ≤ 60 | 14 | 7 | 50.0% | NS | 1 | 7.1% | 3 | 21.4% | 3 | 21.4% | 7 | 50.0% | 6.6 | 0.58 | 0.21 to 1.6 |  |
| Fav. ELN Risk | 1 | 1 | 100.0% | N/A | N/A | N/A | N/A | N/A | 1 | 100% | N/A | N/A | N/A | N/A | N/A |  |
| Int. ELN Risk | 11 | 5 | 45.5% | 1.0000 | 3 |  | 2 |  | 0 |  |  |  | 5.8 | 0.56 | 0.20 to 1.53 |  |
| Adv. ELN Risk | 13 | 7 | 53.8% | NS | 1 |  | 2 |  | 4 |  |  |  | 4.4 | 1.79 | 0.65 to 4.95 |  |

*Supplemental Table 1: Comparisons between patient groups with notable baseline risk factors are again shown, with specific response types also shown. Fisher’s exact test, 2-tailed, was used to calculate for significant difference, and none were seen. Kaplan-Meier survival estimates are shown for these risk groups (right), with logrank calculated hazard ratios also shown. Prior HMA use trended toward significance, but P remained > 0.05, at 0.7. Only a single ELN Favorable Risk patient was treated with survival of 5.5 months for this patient after MLFS response. Due to this limited sample number they were excluded from Fisher’s exact test and logrank analysis.*
